# *N*-Aryl Lactams by Regioselective Ozonation of *N*-Aryl Cyclic Amines

**DOI:** 10.5402/2012/281642

**Published:** 2012-09-18

**Authors:** Francesco Saliu, Marco Orlandi, Maurizio Bruschi

**Affiliations:** Department of Environmental Sciences, University of Milano-Bicocca, Piazza della Scienza 1, 20126 Milano, Italy

## Abstract

Ozonation of *N*-aryl-cyclic amines in organic solvents gave *N*-aryl-lactams regioselectively. In particular, 4-(4-aminophenyl)-morpolin-3-one, a key intermediate in the preparation of factor Xa inhibitors, was obtained in fair yields. The method represents an alternative approach for the lactamization of tertiary *N*-arylic substrates and is based on a “metal-free” introduction of the carbonyl function into the heterocyclic ring.

## 1. Introduction

*N*-aryl lactams are important precursors in the synthesis of alkaloids [1] and of various biologically active compounds. [2] Their chemesthetic activity has been also recently demonstrated [3]. *N*-aryl derivatives of azetidinone have been shown to inactivate human leukocyte elastase (HLE) and porcine pancreatic elastase (PPE) [4]. They are also used as platelet inhibitor [5]. 

**Figure 1:**
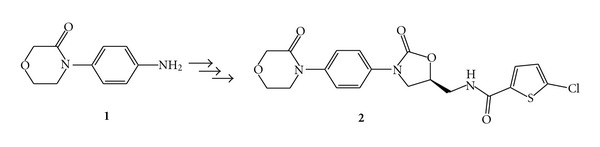
4-(4-aminophenyl)morpholin-3-one (**1**)is a key intermediate for the preparation of 5-chloro-*N*-({(5S)-2-oxo-3-[4-(3-oxo-4-morpholinyl)-phenyl]-1,3-oxazolidin-5-yl}methyl)thiophene-2-carboxamide (**2**).

*N*-aryl lactams relevance in drug development is not just limited to *β*-lactam exponents. For instance, 4-(4-aminophenyl)morpholin-3-one (**1**) is the key precursor (Figure 1) for the preparation of 5-chloro-*N*-({(5S)-2-oxo-3-[4-(3-oxo-4-morpholinyl)-phenyl]-1,3-oxazolidin-5-yl}methyl) thiophene-2-carboxamide (**2**) [6], an inhibitor of coagulation factor Xa used for prophylaxis and treatment of various thromboembolic disorders [7]. 

Moreover, the 4-(4-aminophenyl)morpholin-3-one moiety is present in various factor Xa inhibitors, which entered clinical and preclinical studies [8–12]. 

Two main approaches for the preparation of **1** are described in the literature: the reaction of 2-chloroacetate with anilinoethanol [13], and the metal-mediated selective oxidation at the morpholine ring [14]. The former is preferred on industrial scale due to the cheap reagents employed and the high reactivity exhibited under mild reaction conditions. Nevertheless, processes producing chlorinated waste should be replaced in order to comply with the recommendations of green chemistry. The latter has only an academic perspective at the present time; Markgraf and Stickney recently reported [15] a method for the preparation of *N*-phenyl-lactams based on the selective oxidation of cyclic tertiary amines with potassium permanganate under phase transfer catalysis conditions. The yield in *N*-phenyl-morpholin-3-one achieved from *N*-phenyl-morpholine was 45%. Different results were obtained by using manganese dioxide as oxidant (acetaldehyde and dibutyl acetal were the main products) [16]. 

In our view, the use of ozone could represent an interesting metal-free alternative. Ozonation is widely used in water and wastewater treatment, and it is particularly appealing to reduce trace organic contaminants [17]. The use of ozone in organic solvent for the decomposition of hazardous chemical substances is currently under scrutiny [18]. Some synthetic applications were also proposed: preparation of cycloalkanols and cycloalkanones from cycloalkanes [19], of esters from ethers [20], of amides from amines [21], and of phthalic anhydride from naphthalene [22]. 

Ozonation of aliphatic tertiary amines in water and in various organic solvents is already reported [23, 24]. The products distribution appeared highly influenced by temperature and solvent polarity. Side-chain reactions and *N*-oxide formation were found to occur. Differently, *N*-oxide derivatives were not observed in the ozonation of tertiary aromatic amines [23, 24].

Here we report the results of an experimental investigation about the selective ozonation of tertiary *N*-aryl-cyclic amines for the preparation of *N*-aryl-lactams.

## 2. Results and Discussion

**Figure 2:**
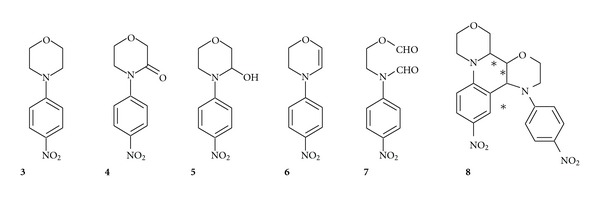
Products deriving from the ozonation of 4-(4-nitrophenyl)morpholine (**3**).

In the first set of experiments 4-(4-nitrophenyl)morpholine (**3**) was reacted with ozone in different solvents and reaction conditions (Figure 2). Very poor yields in the desired 4-(4-nitrophenyl) morpholin-3-one (**4**) were obtained in water (Table 1, entries 9–11). After organic extraction, the unreacted 4-(4-nitrophenyl)morpholine was the main product recovered. A great amount of material (**3**) remains in the aqueous phase, consisting of highly polar degradation products. This is probably due to radical chain reactions, initiated by hydroxyl radical, according to the SBH model[26]. Lactamization was not observed also in butanol (Table 1, entry 13).

Differently, the reactions carried out in organic aprotic solvents (Table 1, entries 1–8) gave 4-(4-nitrophenyl)morpholin-3-one in moderate yields. No appreciable conversions were obtained by using a stream of pure oxygen instead of ozone (Table 1, entry 13).

In particular, conversions were high by using an excess of ozone (Table 1, entries 1-2), but 4-(4-nitrophenyl)morpholin-3-one (**4**) was obtained in moderate yields. Recovery of material after silica gel chromatography was poor, thus showing that more than one-half of the starting reagent had been converted in over-oxidation products, which were retained by silica gel.* 2*-(*N*-formyl-*N*′-4-nitrophenyl-amino)-ethyl formate (**7**) was the main side product, isolated in 1–6% yield. Traces of other reaction products were also observed in the GC-MS chromatograms of the crude reaction mixtures. On the basis of a tentative structural elucidation of the collected mass spectra, they were identified as a dehydro derivative (**6**) of *N*-phenyl-morpholine and dimeric structures (**8**).

**Figure 3:**
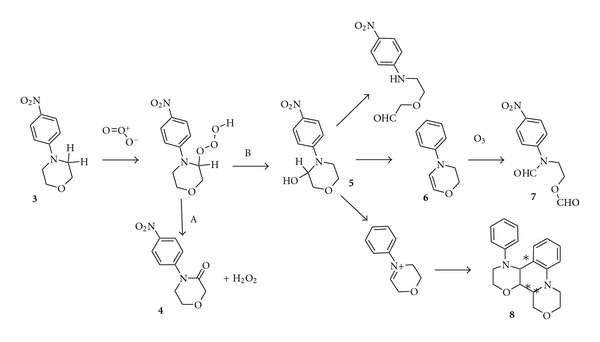
Plausible rearrangement mechanisms of aryl-morpholinyl hydrotrioxide intermediate formed by ozonation of 4-(4-nitrophenyl)morpholine (**3**). Pathway A leads to the formation of the corresponding lactame (**4**), pathway B to dehydro (**6**), diformyl (**7**), and dimeric (**8**) derivates.

Better results were achieved by using a limited amount of ozone; with a ozone/substrate ratio = 2 : 1 in acetonitrile at 0°C for 3h, 4-(4-nitrophenyl)morpholin-3-one was obtained in 54% yield (Table 1, entry 3). Also in acetone the yield was good (Table 1, entry 7). Lower yields were instead obtained by replacing acetonitrile with less polar aprotic solvents such as dichloromethane and hexane (Table 1, entries 5 and 6). These data suggest a solvent polarity effect on the reaction mechanism (Figure 3). In fact, DFT calculations performed on *N*-phenyl-morpholine [27] showed that the activation energy for an hydrogen abstraction in *α* to nitrogen, calculated with respect to the separate reactants, decreases from 7.6 kcal mol^−1^ in *vacuum* to 2.6 kcal mol^−1^ in dichloromethane, to a value as small as equal to 1.6 kcal mol^−1^ in acetonitrile. 

Higher substrate concentration in the reaction media gave higher selectivity in *N*-aryl-lactams but lower conversion. 56% yield in 4-(4-nitrophenyl)morpholin-3-one was obtained (Table 1, entry 15) by adding 20 mmol of 4-(4-nitrophenyl)morpholine to a solution of 100 ml of acetonitrile, kept at 0°C under ozone (5 mg/min) for a total reaction time of 30 minutes. The corresponding 4-(4-aminophenyl) morpholin-3-one was subsequently obtained by regioselective reduction with SnCl_2_ [28]. 

Hence, we submitted this procedure a set of cyclic tertiary amines. *N*-phenylmorpholine gave the corresponding lactame in fair yield (Table 2, entry 1) and a diformyl derivative as main side product. By starting from polymethylenic substrates, selectivities in the corresponding lactames were higher (Table 2, entries 2 and 3). The only side products, detected in trace amounts, were dimeric derivatives. Whereas good yields in 1-phenyl-azetidinone were not achieved (Table 2, entry 4), the method should be considered an interesting alternative to the acylation of anilines because the carbonyl function is directly introduced into the heterocyclic ring after its formation. 

## 3. Conclusion

Ozonation in organic solvent was demonstrated to be a valuable metal-free approach to introduce a carbonyl function on *N*-aryl-cyclic amines. The corresponding *N*-aryl lactams were isolated in fair to good yields. Further investigations are needed in order to increase selectivity. 

## 4. Experimental Section

### 4.1. General

Starting materials were purchased from Sigma-Aldrich. Solvents were solvent grade from Fluka. Compounds **3**, **11**, and **13 **were prepared by Ulmann literature method [29]. Compound **16  **was purchased from Acros Organic. Compound **16** was**  **prepared by cyclization of the corresponding propionyl halogenated amide [3], and used as reference material. Ozonations were performed with a corona-discharge-based ozone generator (by Ozono Elettronica Internazionale s.r.l) using oxygen as feed gas (30 L/h of a gas stream). The gas diffusion in the liquid phase was obtained through a Pasteur pipette 2 mm wide. GC-MS analyses were performed with a Hewlett Packard 5890A instrument, (split/splitless injector, capillary column SPB-5, 30 m, 0.32 mm I.D.) equipped with a 5971A mass selective detector. ^1^H-NMR spectra were recorded in CDCl_3_ on a Varian Mercury 400 instrument. Chemical shifts were reported in parts per million (*δ*), relative to the internal standard of tetramethylsilane (TMS). Infrared (IR) spectra in solutions were recorded on a Nicolet Avatar 360 Fourier transform (FT)-IR spectrometer, using calcium fluoride cells.

### 4.2. Representative Experimental Procedure for Oxidation of *N*-Aryl-Lactam

20 mmol of the selected amines were added to a solution of 100 ml of acetonitrile, kept at 0°C under a continuous stream of ozone (5 mg/min) for a total reaction time of 30 minutes. The residue was evaporated under a nitrogen stream and extracted with alkaline water. Products were isolated by flash chromatography over silica gel (*R* = 100), by eluting with a mixture of dichloromethane-ethyl acetate 9 : 1. ^1^H-NMR, IR, and mass spectra of compounds **10**, **12**, and **14 **were in accordance with literature data [15]. 

*4-(4-nitrophenyl)-3-morpholinone *(**4**) had IR (CH_2_Cl_2_): 1665 cm^−1^, MS: *m/z* = 222 (M^+^), 193 (M^+^–CHO), 164 (M^+^–2CHO), 150 (M^+^–2CHO–CH3), 136, 120, 106, 90, 76, 68, 52; ^1^H-NMR:  *δ* = 3.07 (*t*, *J* = 5.14 Hz, 2H), 3.97 (*t*, *J* = 5.14 Hz, 2H), 4.28 (s, 2H), 7.20–7.40 (m, 2H); 7.75–7.95 (m, 2H); ^13^CNMR:  *δ* = 163.0, 142.4, 126.5, 124.0, 68.3, 66.1, 43.5. 

*2-(N-formyl-N* ^'^ *-4-nitrophenyl-amino)-ethyl formate* (**7**) had IR (CH_2_Cl_22_): 1729, 1671 cm^−1^, MS: *m*/*z* = 238 (M^+^), 210 (M^+^–CO), 192 (M^+^–HCOOH), 181 (M^+^–CO–CHO) _1_H-NMR: *δ* = 4.03 (*t*, *J* = 5.12 Hz, 2H), 4.27 (*t*, *J* = 5.12 Hz, 2H), 7.20–7.40 (m, 2H), 7.75–7.95 (m, 2H), 7.91 (s, 1H), 8.34 (s, 1H); ^13^C-NMR: *δ* = 161.0, 160.0, 141.0, 126.5, 124.7, 60.3, 44.1. 

*1-(4-nitrophenyl)-azetid-3-one* (**16**) had IR (CH_2_Cl_2_): 1734 cm^−1^, MS: *m/z* = 177 (M^+^), 148 (M^+^–CHO), 119 (M^+^–2CHO), 104 (M^+^–2CHO–CH3) 91, 77, 63, 51; ^1^H-NMR: *δ* = 3.13 (*t*, *J* = 1.65 Hz, 3H), 3.68 (*t*, *J* = 1.67 Hz, 4H), 7.10–7.45 (m, 5H).

## Supplementary Material

INSTRUMENT: HP 5973A Mass Selective Detector, HP5890 series II Splitless Capillary Inlet Flow , manual injection. Capillary Column lenght 30 meters, I.D. 0,35 mm, film 0.25 *µ*m, phase (5%-Phenyl)-methylpolysiloxane equivalent to USP Phase G27, 2ml/min He solvent CH_2_Cl_2_ stabilized with amylene.

## Figures and Tables

**Table 1:** Conversion and isolated yields in 4-(4-nitrophenyl)-morpholin-3-one (**4**) after ozonation of 4-(4-nitrophenyl)-morpholine (**3**) at different experimental conditions (DCM: dichloromethane, CAN: acetonitrile, EA: ethyl acetate).

Entry	*T* (°C)	*t* (h)	Solvent	Ozone^a^	Conversion^b^ (%)	**4** (%)^c^	Other
1	25	1.5	ACN	excess	100	18	6, 7, 8
2	0	1	ACN	excess	100	35	6, 7, 8
3	0	3	ACN	2 : 1	100	54	7, 8
4	0	3	ACN	1 : 10	11	8	—
5	0	3	DCM	2 : 1	91	29	7, 8
6	0	3	n-Hexane	2 : 1	80	24	6, 8
7	0	3	Acetone	2 : 1	100	52	6, 7, 8
8	0	3	EA	2 : 1	98	34	**7**
9	5	0.5	Water PH = 7	excess	87	2	—
10	5	3	Water pH = 3	8 : 1	74	1	—
11	5	3	Water pH = 12	8 : 1	81	0	—
12	5	3	n-Butanol	6 : 1	72	2	—
13	0	3	ACN	none	2	0	—
15^d^	0	0.5	ACN	1 : 40	74	56	7, 8

^a^100 mg of 4-(4-nitrophenyl)-morpholine (**3**) were dissolved in 50 mL of solvent and ozonized at the selected temperature for the selected time. When the reactions were carried out with a limited amount of ozone, 30 mg 4-(4-nitrophenyl)morpholine (**3**) were dissolved in the required amount of solvent. Ozone concentrations were estimated on the basis of the iodometric method [25].

^b^Conversions are calculated on the recovered 4-(4-nitrophenyl)morpholine.

^c^GC-MS yield.

^d^For the conditions see the experimental section.

**Table 2:** Synthesis of *N*-aryl-lactams by ozonation of *N*-*N-*polymethylene-anilines.

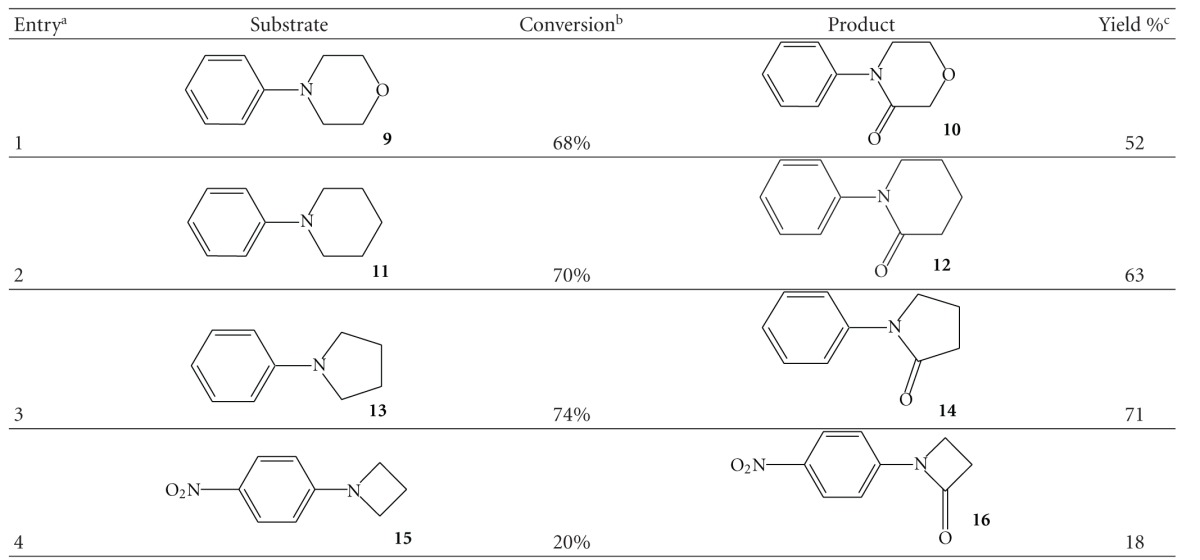

^a^For the condition see the experimental part.

^b^Conversions are calculated on the recovered starting material.

^c^Isolated yield after silica gel chromatography.
