# Supplementary material for: N-Aryl Lactams by Regioselective Ozonation of N-Aryl Cyclic Amines
Source: ISRN Org Chem. 2012 Sep 18;2012:281642. doi: 10.5402/2012/281642 (PMC3767345; doi:10.5402/2012/281642)
Supplement: Supplementary file 1 — INSTRUMENT: HP 5973A Mass Selective Detector, HP5890 series II Splitless Capillary Inlet Flow , manual injection. Capillary Column lenght 30 meters, I.D. 0,35 mm, film 0.25 µm, phase (5%-Phenyl)-methylpolysiloxane equivalent to USP Phase G27, 2ml/min He solvent CH2Cl2 stabilized with amylene. [file 281642.f1.doc]

**SUPPLEMENTARY DATA**

**MS spectra of the selected compounds**

INSTRUMENT: HP 5973A Mass Selective Detector, HP5890 series II Splitless Capillary Inlet Flow , manual injection. Capillary Column lenght 30 meters, I.D. 0,35mm, film 0.25 µm, phase (5%-Phenyl)-methylpolysiloxane equivalent to USP Phase G27, 2ml/min He

solvent CH_2_Cl_2_ stabilized with amylene
